# Association of childhood bullying victimisation with suicide deaths: findings from a 50-year nationwide cohort study

**DOI:** 10.1017/S0033291722000836

**Published:** 2023-07

**Authors:** Marie-Claude Geoffroy, Louise Arseneault, Alain Girard, Isabelle Ouellet-Morin, Chris Power

**Affiliations:** 1 Departments of Psychiatry and Educational and Counselling Psychology, McGill University, Montreal, Quebec, Canada; 2 Institute of Psychiatry, Psychology and Neuroscience, King's College London, London, UK; 3 CHU Sainte-Justine Research Center, Montreal, Canada; 4 School of Criminology, University of Montreal, Montreal, Quebec, Canada; 5 UCL Great Ormond Street Institute of Child Health, London, UK

**Keywords:** Bullying victimisation, lifecourse, risk factors, suicide

## Abstract

**Background:**

Bullying victimisation has been associated with increased risk of suicide ideation and attempt throughout the lifespan, but no study has yet examined whether it translates to a greater risk of death by suicide. We aimed to determine the association of bullying victimisation with suicide mortality.

**Methods:**

Participants were drawn from the 1958 British birth cohort, a prospective follow-up of all births in 1 week in Britain in 1958. We conducted logistic regressions on 14 946 participants whose mothers reported bullying victimisation at 7 and 11 years with linked information on suicide deaths through the National Health Service Central Register.

**Results:**

Fifty-five participants (48 males) had died by suicide between the age 18 and 52 years. Bullying victimisation was associated with suicide mortality; a one standard deviation increases in bullying victimisation linked to an increased odds for suicide mortality [odds ratio (OR) 1.29; 1.02–1.64] during adulthood. The OR attenuated by 11% after adjustment for individual (e.g. behavioural and emotional problems) and familial characteristics (e.g. adverse childhood experiences, 1.18; 0.92–1.51). Analysis of bullying victimisation frequency categories yields similar results: compared with individuals who had not been bullied, those who had been frequently bullied had an increased odds for suicide mortality (OR 1.89; 0.99–3.62).

**Conclusion:**

Our study suggests that individuals who have been frequently bullied have a small increased risk of dying by suicide, when no other risk factors is considered. Suicide prevention might start in childhood, with bullying included in a range of inter-correlated vulnerabilities encompassing behavioural and emotional difficulties and adverse experiences within the family.

## Introduction

Suicide is one of the leading causes of death across the life-course, with 700 000 deaths each year around the world (Glenn et al., 2020; Naghavi, 2019; Turecki & Brent, 2016; World Health Organization, 2021). Every suicide is a tragedy affecting not only the person who died by suicide, but also networks of people who knew the deceased and who might be in need of support (Cerel et al., 2019).

While suicide is a multifactorial phenomenon involving complex interactions between a range of risk and protective factors (Turecki & Brent, 2016), one factor that has attracted considerable public attention recently is peer and bullying victimisation (Moreno, Gower, Brittain, & Vaillancourt, 2019). Peer and bullying victimisation are the experience of being the target of the aggressive behaviour by others from the same age group (Finkelhor, Turner, & Hamby, 2012; Hawker & Boulton, 2000). It can include being pushed or hit, being called names, and made fun of, or being excluded from a group. Bullying is a specific form of peer victimisation which is characterised by an imbalance of power between the perpetrator and the victim, as well as repetition of the acts (Olweus, 1993). While children from diverse cultural and geographic backgrounds are exposed to peer and bullying victimisation (World Health Organisation, 2012), not all children are equally likely to be exposed to such experiences. Vulnerable children such as those with lower IQ or disruptive behaviours (Oncioiu et al., 2020), those raised in an abusive home (Lereya, Samara, & Wolke, 2013) and/or who carry a range of genetic vulnerabilities related to these individual characteristics (Schoeler et al., 2019) are more likely to become the victims of their peers.

Prior systematic reviews and meta-analyses and recent observational and quasi-experimental studies found that exposure to bullying victimisation in childhood predicted suicidal ideation and suicide attempts in adolescence and early adulthood (Castellví et al., 2017; Geoffroy et al., 2018a, 2016; Herba et al., 2008; Holt et al., 2015; John et al., 2018; Moore et al., 2017; O'Reilly et al., 2021; Schoeler, Duncan, Cecil, Ploubidis, & Pingault, 2018). A previous study from the 1958 British birth cohort reported that individuals who were frequently bullied had higher rates of suicidal ideation in their forties (individuals who reported that life is not worth living or who thought about suicide) than those who had not been bullied (Takizawa, Maughan, & Arseneault, 2014), suggesting that bullying victimisation could act as an early adverse experience placing an individual at risk of later suicide death. Findings from the Finnish 1981 birth cohort study (Klomek et al., 2009), indicated that being the victim of bullying at 8 years was associated with suicide attempts that required hospital admission (*n* = 42) and suicide mortality (*n* = 15, 2 females) after statistically controlling for conduct and depression symptoms at 8 years [odds ratio (OR) 6.3 (1.5–25.9) in females and 3.8 (0.99–14.3) in males]. However, as suicide attempts and deaths were combined, the specific association with suicide deaths remained unknown. While studies so far suggest that childhood bullying victimisation may predispose an individual to suicide death, possibly via an association with ideation and attempt, no studies have yet examined the prospective association of childhood bullying victimisation with suicide mortality.

Although suicide ideation, suicide attempts and suicide mortality share a range of common risk factors, specific risk factors have also been identified (Klonsky, Saffer, & Bryan, 2018; Mars et al., 2019). For example, early adversity, including peer bullying victimisation and abusive home, was not associated with the transition to suicide attempts among individuals who previously experienced suicidal ideation (Mars et al., 2019). Insights into the mechanisms leading to suicide may be gained by establishing associations of childhood bullying victimisation with suicide mortality in adulthood.

Using the information on bullying victimisation in childhood and suicide mortality in adulthood collected by the 1958 British birth cohort, we investigated whether being a victim of bullying in childhood was associated with suicide mortality in adulthood. Pre-existing vulnerabilities linked to later suicide mortality such as adverse childhood experiences (ACEs) within the family and emotional and behavioural problems are risk factors for exposure to bullying (Arseneault, 2018; Oncioiu et al., 2020) and they may either confound the unique association between childhood bullying victimisation and suicide mortality or exacerbate suicidal risk prior to or following exposure to bullying victimisation. Our analyses tested (1) the associations between childhood bullying victimisation and suicide death up to mid-life; (2) whether the associations persisted after accounting for sex and a range of childhood confounders including adverse intra-familial experiences and emotional and behavioural problems; and (3) whether suicide risk associated with bullying victimisation was exacerbated by ACEs and childhood emotional and behavioural problems (Afifi et al., 2020; Fisher et al., 2012; Zhu et al., 2016).

## Methods

### Participants

The 1958 British birth cohort, also known as the National Child Development Study, is an ongoing longitudinal national birth cohort of 17 638 individuals born in 1 week of March 1958 in England, Scotland, and Wales and representing 98% of all births in Britain in that week. Details about the cohort, including study design and response rates can be found elsewhere (Power & Elliott, 2006).

Of the 17 638 participants in the birth survey of 1958, we excluded 1168 participants who had emigrated permanently from Britain (up to 2009, as information was not available thereafter) because the National Health Service Central Register is not notified of deaths of emigrants. We further excluded 1524 participants who had died from causes other than suicide by 2012 (most deaths had occurred within the first year of life), leaving 14 946 participants for the analyses on suicide deaths.

Ethical approval was given by the South-East Multicentre Research Ethics Committee and written consent was obtained from all participants.

## Measures

### Childhood bullying victimisation

When participants were 7 and 11 years, mothers were asked about the frequency their child had been bullied by other children: ‘never’, ‘sometimes’ or ‘frequently’. We created a continuous scale by summing participants' scores across the two time points to reflect the frequency of bullying victimisation at both ages. We transformed the scale into Z-scores to ease interpretation (ranging from −0.72 to 3.49 s.d.). While the continuous bullying victimisation frequency score was used as exposure in our main analyses, we also used a categorical variable for consistency across studies on bullying victimisation in this cohort (Brimblecombe et al., 2018; Evans-Lacko et al., 2017; Takizawa, Danese, Maughan, & Arseneault, 2015; Takizawa et al., 2014). Accordingly, we used a three-level childhood bullying victimisation indicator based on responses at both time points: 0 = not been bullied (never at both 7 and 11 years); 1 = occasionally (sometimes at either age); and 2 = frequently bullied (frequently at either age or sometimes at both ages).

### Suicide mortality

Participants were linked with death records via the National Health Service Central Register (NHSCR); until 31 August 2012 (end of mortality follow-up year). Similarly to our prior publications on suicidality with this cohort (Geoffroy, Gunnell, & Power, 2014; Richard-Devantoy et al., 2021), suicides were identified using the International Classification of Diseases, ninth revision (ICD-9) codes E950–59 (suicide) and E980–89 (undetermined intent) or tenth revision (ICD-10) codes X60–84 (suicide) and Y10–34 (undetermined intent). Suicide and death of undetermined intent were combined (Gunnell et al., 2013). We excluded pending verdicts (ICD-9 code 988.88; ICD-10 code Y33.9). Age at death by suicide was extracted from death certificates and ranged from 18 to 52 years.

### Potential confounders

A range of childhood individual and family characteristics were selected based on the literature which includes prior studies with the cohort for their associations with bullying victimisation and suicide mortality (Evans-Lacko et al., 2017; Geoffroy, Gunnell, Clark, & Power, 2018b; Geoffroy et al., 2014; Richard-Devantoy et al., 2021; Takizawa et al., 2015, 2014).

#### Individual factors

Childhood emotional and behavioural problems (e.g. miserable, resentful/aggressive) were reported at 7 years by the teachers via the 146-item Bristol Social Adjustment Guide (BSAG) (Stott, 1969). Childhood cognitive abilities were assessed via tests of reading (word recognition Southgate test) (Southgate, 1962) and arithmetic, consisting of 10 problems of graded difficulty administered to participants at age 7 years. The two tests were standardised for month and year of assessment and averaged to obtain a summary score.

#### Family factors

Childhood socioeconomic disadvantage was identified from information on father's social class at birth [grouped as class I or II (professional/managerial), IIINM (skilled non-manual), IIIM (skilled manual) and IV and V (semi-unskilled manual, including single households)], household amenities (bathroom, indoor lavatory, hot water) and household crowding at 7 years. Maternal age at birth was obtained from medical records. ACEs within the family by age 7 years were extracted following a procedure described in prior studies with this cohort (Barboza Solís et al., 2015) and identified as a set of stressful familial psychosocial conditions. After the age-7 interview with parents in the participant's home, health visitors recorded information on whether (1) the child has ever been in public/voluntary care services or foster care; the child lived in a household where a family member (2) is in contact with probation service; (3) has mental illness/has contact with mental health service; (4) has alcohol abuse problem; (5) the child has been separated from their father or mother due to death or divorce, or separation. The child's schoolteacher at 7 years reported (6) whether the child appears undernourished or dirty. Exposure to ACEs was identified by an affirmative response to any of the six categories.

### Statistical analyses

We examined bivariate associations between each individual and familial characteristic and suicide mortality and childhood bullying. Then, we tested whether the frequency of childhood bullying victimisation (continuous score) was associated with suicide mortality using logistic regressions. We estimated four models (1) unadjusted, (2) sex-adjusted, (3) additionally adjusted for individual characteristics, including childhood emotional and behavioural problems, and cognitive skills; and (4) additionally adjusted for familial characteristics including family socioeconomic disadvantage, maternal age at birth and ACEs. We also tested whether associations of bullying victimisation differed by childhood emotional and behavioural problems and by the total number of ACEs. To do so, we estimated two separate models where bullying and (1) emotional and behavioural problems and (2) ACEs, as well as the two-ways interactions between those variables (bullying × emotional and behavioural problems; bullying × ACEs) were used as predictors of suicide mortality. We tested whether bullying victimisation was associated with age at death by suicide. We used linear regressions with age at death by suicide as the outcome and bullying victimisation as the exposure. Finally, we re-estimated our models using bullying categories as the exposure variable (none, occasionally and frequently).

Missing data on bullying victimisation ranged from 13.4% at 7 years to 18.3% at 11 years (e.g. 6.1% of children were missing at both times including one suicide) while for confounders the range was from 0% (sex) to 12.1% (cognitive ability). To minimise data loss, missing data on both childhood bullying victimisation and confounding variables were imputed using multiple imputation chained equations (Azur, Stuart, Frangakis, & Leaf, 2011) with all variables in the models, together with participants' mental health at 16 years, which has been shown to be a key predictor of missingness. Regression analyses were run across 100 imputed datasets. Imputed results were broadly similar to those using observed values (online Supplementary Table S1); the former is presented. Statistics were conducted in SPSS version 26th.

### Sensitivity analysis

To complement our inferential statistical approach, and because the analyses relied on a very low frequency of suicide, we applied a Bayesian statistical approach (Schmalz, Biurrun Manresa, & Zhang, 2021) to quantify evidence for the alternative (H1) relative to the null (H0) hypotheses using R version 4.1.2 (R Core Team, 2020) with package brms (Bürkner, 2017) and bayestestR (Makowski, Ben-Shachar, & Lüdecke, 2019). A Bayes factor (BF10) is the ratio between the evidence that supports an association of bullying victimisation with suicide (H1) over the evidence supporting no association between bullying victimisation and suicide (H0). BFs greater than 1 usually indicate relative evidence for H1, whereas those smaller than 1 indicate relative evidence for H0. BFs ranging between 1–3 and 3–10 suggest anecdotal and moderate evidence for H1, respectively, whereas a BF greater than 10 represents strong evidence for H1 (Aczel, Palfi, & Szaszi, 2017). BFs were calculated using bridge-sampling (Gronau, Singmann, & Wagenmakers, 2020). All models were evaluated using non-informative priors [log student-t (3, 0, 2.5)], which are the default priors in brms.

## Results

Fifty-five participants had died from suicide (0.36%; 48 males and 7 females) by age 52 years; the median age of suicide was 41 years (range 18–52 years) for men and 44 years (range 21–49 years) for women.

Table 1 shows Pearson correlations for suicide mortality and potential confounders and online Supplementary Table S2 Pearson correlations between potential confounders. Individuals who died by suicide had a pattern of greater levels of individual and familial vulnerabilities than their counterparts. In addition, individual and familial characteristics were correlated with childhood bullying victimisation (Table 2).

**Table 1.** Descriptive statistics (mean, s.e. or %, *N*) for suicide mortality in adulthood by childhood individual and family factors^±^

	Suicide death from 18 to 52 years		
	Yes (*N* = 55)	No (*N* = 14 891)	*p* Value^a^
Individual characteristics			
Sex (male), %, *n*	87.3 (48)	50.7 (7543)	<0.001
Emotional and behavioural problems (range 0 to 64)	12.28 (1.23)	9.10 (0.07)	0.010
Cognitive ability (range −4.04 to 1.73)	0.01 (0.14)	−0.02 (0.01)	0.797
Familial characteristics			
Socioeconomic disadvantage (range 1 to 12)	7.78 (0.34)	7.27 (0.02)	0.137
Maternal age at child's birth (range 14 to 47)	26.00 (0.65)	27.51(0.05)	0.021
Adverse childhood experiences (range 0 to 6)	0.35 (0.09)	0.21 (0.004)	0.135

± Based on imputed data (*n* = 14 946 for suicide mortality).

a Based on *t* tests or χ^2^.

**Table 2.** Correlations between childhood individual and family factors and bullying victimisation^±^

	Childhood bullying victimisation
Individual characteristics	
Sex (male)	**0.07**
Emotional and behavioural problems	**0.17**
Cognitive ability	**−0.17**
Family characteristics	
Socioeconomic disadvantage	**0.10**
Maternal age at birth	**−0.07**
Adverse childhood experiences	**0.12**

Based on Pearson correlations. Bolded estimates are statistically significant; *p* < 0.05.

± Based on imputed values, *n* = 14 946.

Table 3 shows OR and 95% confidence intervals (CI) for the association of childhood bullying victimisation score with suicide mortality. In unadjusted models, the bullying victimisation scale was associated with increased odds of suicide mortality (OR 1.29; 95% CI 1.02–1.64). That is, every standard deviation increases in the bullying victimisation scale was associated with a 29% increase in the odds of suicide mortality. The odds attenuated by 7% after controlling for sex (OR 1.23; 95% CI 0.97–1.57) and by 4% further after accounting for individual and familial vulnerabilities (1.18; 95% CI 0.92–1.51). We found no evidence for a moderating effect of childhood emotional and behavioural problems or ACEs (*p* values for interaction terms were 0.870 and 0.743 respectively). Additional analyses with an age of death by suicide as the outcome indicated that bullying victimisation was not associated with age at death by suicide (unadjusted *ß* = 0.25, s.e. = 1.15; *p* = 0.826; fully adjusted (model 4) *ß* = 0.434, s.e. = 1.23; *p* = 0.725).

**Table 3.** OR (95% CI) for the association of childhood bullying victimisation with suicide mortality by mid-adulthood^±^

	OR (95% CI) for suicide death	*p* Values
Childhood bullying victimisation		
Unadjusted	1.29 (1.02–1.64)	0.032
Adjusted for sex	1.23 (0.97–1.57)	0.082
Adjusted for individual factors^a^	1.22 (0.95–1.55)	0.112
Adjusted for family factors^b^	1.18 (0.92–1.51)	0.200

OR, odds ratio; 95% CI, 95% confidence intervals.

± Based on imputed values (*n* = 14 946).

a The model is adjusted for sex and individual characteristics including emotional and behavioural problems, and cognitive ability.

b The model is adjusted for sex, individual characteristics including emotional and behavioural problems, and cognitive ability and family characteristics including socioeconomic disadvantage, maternal age and adverse childhood experiences.

Further, analyses of bullying victimisation as a categorical exposure showed that frequent bullying victimisation was marginally associated with an increased risk of suicide death, although CIs were wide due to low prevalence of suicide (Table 4). Associations reduced after adjustment for individual and familial vulnerabilities: OR for suicide death from 1.89 (0.99–3.62) to 1.45 (0.74–2.83).

**Table 4.** OR (95% CI) for the association of frequent and occasional childhood bullying victimisation with suicide mortality by mid-adulthood^±^

Childhood bullying frequency categories	Suicide death^†^ %(*n*)		OR (95% CI) for Suicide death^±^	*p* Values
	No	Yes		
Unadjusted				
Not bullied	54.1 (6073)	43.2 (19)	Ref.	
Occasionally	27.9 (3135)	22.7 (10)	0.98 (0.47–2.04)	0.953
Frequently	18.0 (2023)	34.1 (15)	1.89 (0.99–3.62)	0.054
Adjusted for sex				
Occasionally			0.92 (0.44–1.91)	0.816
Frequently			1.67 (0.87–3.19)	0.124
Adjusted for individual factors^a^				
Occasionally			0.90 (0.43–1.89)	0.783
Frequently			1.60 (0.83–3.11)	0.163
Adjusted for family factors^b^				
Occasionally			0.86 (0.41–1.80)	0.430
Frequently			1.45 (0.74–2.83)	0.826

OR, odds ratio; 95% CI, confidence intervals.

± Based on imputed values (*n* = 14 946).

† Prevalence and *n* are based on observed data.

a The model is adjusted for sex and individual characteristics including emotional and behavioural problems, and cognitive ability.

b The model is adjusted for sex, individual characteristics including emotional and behavioural problems, and cognitive ability and family characteristics including socioeconomic disadvantage, maternal age and adverse childhood experiences.

Finally, as shown in Table 5, BFs yield moderate evidence in favour of H1 for the unadjusted model and anecdotal evidence in models adjusting for other childhood individual and familial risk factors pertaining to suicide.

**Table 5.** Bayes factors calculated for the association between childhood bullying victimisation and suicide mortality by mid-adulthood^±^

	BF_10_	Interpretation
Childhood bullying victimisation		
Unadjusted	3.73	Moderate evidence in favour of H_1_ *v*. H_0_
Adjusted for sex	1.80	Anecdotal evidence in favour of H_1_ *v*. H_0_
Adjusted for individual factors^a^	1.35	Anecdotal evidence in favour of H_1_ *v*. H_0_
Adjusted for family factors^b^	0.877	Anecdotal evidence in favour of H_0_ *v*. H_1_

OR, odds ratio; 95% CI, 95% confidence intervals.

± Based on completed case (*n* = 12 661).

a The model is adjusted for sex and individual characteristics including emotional and behavioural problems, and cognitive ability.

b The model is adjusted for sex, individual characteristics including emotional and behavioural problems, and cognitive ability and family characteristics including socioeconomic disadvantage, maternal age and adverse childhood experiences.

## Discussion

Bullying and peer victimisation have been associated with suicidal ideation and suicide attempts in adolescence (Geoffroy et al., 2018a, 2016; Gini & Espelage, 2014; Holt et al., 2015; Moore et al., 2017; Perret et al., 2020) and adulthood (Copeland, Wolke, Angold, & Costello, 2013; Klomek et al., 2009; Takizawa et al., 2014). Our study shows, in a 5-decade nationwide prospective cohort, that individuals who have been frequently bullied in childhood had an increased, albeit small, risk of dying by suicide in the adult years, when no other risk factors for suicide were considered.

To our knowledge this is the first prospective study to examine the role of childhood bullying victimisation on later suicide mortality. One prior study by Klomek et al. (2009) combining information on hospital admission for suicide attempts and mortality by age 25 found an association of frequent bullying victimisation. However, as suicide attempts and deaths were combined with a low number of suicides (*n* = 15 in total; *n* = 13 in males) the specific associations with suicide deaths were unknown. The 1958 British birth cohort enabled us to capture a larger group of suicide deaths. A small association of childhood bullying victimisation and suicidal mortality throughout the adult years was detected, which reduced by 11% after confounding factors were controlled for. This pattern of association was consistent across both continuous and categorical measures of bullying victimisation. A prior study in this cohort of the association between frequent bullying (*v.* not bullied) and suicidality at 45 years reported an adjusted OR of 1.66 (Takizawa et al., 2014). This estimate is comparable to the OR of 1.45 reported in our study. While both studies allowed for a similar range of confounding factors, the study by Takizawa et al. (2014) was on suicidality at age 45 years (defined as ‘thought that life is not worth living’ and ‘thought of killing yourself in the past week’) with a higher statistical power than our study of suicide deaths.

Our findings do not support the assumption that pre-existing individual (childhood emotional and behavioural problems) and familial (adversity) vulnerabilities could exacerbate suicidal risk following bullying victimisation, although this finding warrants replication given the low number of suicide deaths (and related lower power to detect such effect). Our findings nevertheless point towards a cumulative effect of bullying with other forms of adversity (Afifi et al., 2020). Indeed, our study suggests that individuals exposed to the highest levels of bullying victimisation were also exposed to other forms of adverse experiences in their family, hence cumulating risk factors for suicide. Further, while prior studies have shown that there is a continuity of victimisation (including bullying victimisation) across ages (Finkelhor, Ormrod, & Turner, 2007), our measure of bullying victimisation only captures two time points in childhood. Victimisation may have continued into later life (i.e. re-victimisation and poly-victimisation). Altogether, interventions that contribute to break this vicious cycle of violence victimisation may help to reduce the risk for suicide.

In this cohort, most suicides occurred in the early forties and while we did not detect an association between childhood bullying victimisation and age at the time of death, we can not rule out the possibility that bullying victimisation might increase suicide risk factors that are in closer temporal proximity, that is, during adolescence or emerging adulthood. Of note, interpersonal conflicts and relationship difficulties are often cited as key precipitants of suicide death in psychological autopsy studies (Marttunen, Aro, & Lönnqvist, 1993) and bullying victimisation was reported in about one-quarter of suicides in people younger than 20 years (Rodway et al., 2016). However, in an observational study based on 94 youth suicides in Canada, bullying had been mentioned in coroners' verdicts for a few suicides only, with more common proximal stressors mentioned being conflict with parents or romantic partners (Sinyor, Schaffer, & Cheung, 2014). Further, recent studies indicate that the new form of bullying victimisation, cyberbullying, is more strongly associated, in the short term, with suicidal ideation and attempts than traditional or face-to-face bullying (Gini & Espelage, 2014; Perret et al., 2020). Studies with measures of all forms of bullying, including cyberbullying, and records of suicide by early adulthood are needed to further build the evidence base for the association of bullying victimisation and suicide mortality.

### Methodological considerations

Our study population is large and nationally representative of British residents born in 1958, and captures for the first time, prospectively collected information on childhood bullying victimisation and suicide mortality over five decades of life. However, our results should be interpreted in light of some limitations. First, our study may be underpowered to detect an increasing risk of death by suicide, a rare phenomenon, among individuals who were bullied in childhood. For our bullying victimisation score, CI were wide, i.e. such as 1.02 to 1.64 in the unadjusted model, suggesting that the estimated risk ranges between 2% and 64% increase in suicide mortality (or between 8% decrease to 51% increase when adjusting for all potential confounding factors). The estimation of BFs similarly provides moderate evidence for an association of bullying victimisation with suicide mortality, and anecdotal evidence for rejecting the alternative hypothesis after controlling for other childhood risk factors for suicide. Second, while information on a set of key potential confounding factors was available, some factors (e.g. cognitive ability and childhood emotional and behavioural problems) were measured concurrently with bullying victimisation. Because bidirectional associations exist between bullying victimisation and these individual characteristics (Brunstein Klomek et al., 2019), we cannot exclude the possibility of inverse causality, and thereby to a risk of over-adjustment in our models. Accordingly, the inclusion of these confounders may have reduced the power to detect an association. between bullying and mortality. Third, our bullying victimisation measure was based on a single question administered to mothers (was your child ever bullied by other children), albeit at two separate ages, and no definition of bullying was provided. As parents may not be aware of all experiences of bullying victimisation (Stockdale, Hangaduambo, Duys, Larson, & Sarvela, 2002), their occurrence might have been underreported by mothers, hence leading to misclassification of some children into the ‘not bullied’ category, which could have further reduced power. Despite these limitations in measurement, prior studies based on the 1958 British birth cohort reported consequences of bullying victimisation on a range of outcomes including midlife mental health (Evans-Lacko et al., 2017; Takizawa et al., 2014), income (Brimblecombe et al., 2018; Takizawa et al., 2015) and inflammatory markers, supporting the construct validity of the bullying measure. Furthermore, reports of bullying victimisation from both mothers and children have been similarly associated with children's emotional and behavioural problems (Shakoor et al., 2011). Fourth, while the cohort captures all births in a single week of March, it does not include children born in the summer or who are young for their school year, and who may be more often targeted due to their greater developmental immaturity (Whitely et al., 2019).

## Conclusion

Although firm conclusions can't be drawn based on this study alone, our results suggest that childhood bullying victimisation may carry a small risk for suicide deaths in adulthood. However, larger population-based studies are needed to confirm these results and to clarify whether childhood bullying has a separate role beyond other childhood risk factors for death by suicide decades later. Nevertheless, suicide prevention strategies are often recommended to start in childhood, with bullying included in a constellation of co-occurring vulnerabilities encompassing behavioural and emotional difficulties, as well as adverse experiences within the family.

## Supplementary material

For supplementary material accompanying this paper visit https://doi.org/10.1017/S0033291722000836. click here to view supplementary material
