# Supplementary material for: Association of childhood bullying victimisation with suicide deaths: findings from a 50-year nationwide cohort study
Source: Psychol Med. 2022 Apr 7;53(9):4152–9. doi: 10.1017/S0033291722000836 (PMC10317807; doi:10.1017/S0033291722000836)
Supplement: Supplementary file 1 [file S0033291722000836sup001.docx]

**Supplemental Table 1.** OR (95% CI) for the association of childhood bullying victimization with suicide mortality by mid-adulthood, complete case^±^

|  | OR (95% CI) for suicide death | P-Values |
| --- | --- | --- |
| **Childhood bullying victimization** |  |  |
| Unadjusted | 1.32 (1.05, 1.67) | .017 |
| Adjusted for sex | 1.27 (1.00, 1.60) | .047 |
| Adjusted for individual factors^a^ | 1.24 (0.98, 1.58) | .073 |
| Adjusted for family factors^b^ | 1.20 (0.94, 1.53) | .135 |

OR=odds ratio; 95%CI= 95% confidence intervals.
^±^ Based on comleted case (*n*=12 661)

^a^The model is adjusted for sex and individual characteristics including emotional and behavioural problems, and cognitive ability.

^b^The model is adjusted for sex, individual characteristics including emotional and behavioural problems, and cognitive ability and family characteristics including socioeconomic disadvantage, maternal age and adverse childhood experiences.

**Supplemental Table 2.** Correlations between potential confounding factors.^±^

|  | Sex (male) | Emotional and behavioural problems | Cognitive ability | Socio  economic disadvantage | Maternal age at birth | Adverse childhood experiences |
| --- | --- | --- | --- | --- | --- | --- |
| Sex (male) | 1.00 |  |  |  |  |  |
| Emotional and behavioural problems | .15*** | 1.00 |  |  |  |  |
| Cognitive ability | -.01 | -.41*** | 1.00 |  |  |  |
| Socioeconomic disadvantage | -.01 | .15*** | -.25*** | 1.00 |  |  |
| Maternal age at birth | -.01 | -.02** | .03** | -.13*** | 1.00 |  |
| Adverse childhood experiences | .01 | .22*** | -.19*** | .16*** | -.02 | 1.00 |

±Based on imputed values, n= 14,946

Based on Pearson correlations

*** Correlation is significant at the .001 level (2-tailed)

** Correlation is significant at the .01 level (2-tailed)
